# Green Extraction of Natural Antioxidants from the *Sterculia nobilis* Fruit Waste and Analysis of Phenolic Profile

**DOI:** 10.3390/molecules23051059

**Published:** 2018-05-02

**Authors:** Jiao-Jiao Zhang, Ya Li, Sheng-Jun Lin, Hua-Bin Li

**Affiliations:** 1 Guangdong Provincial Key Laboratory of Food, Nutrition and Health, Department of Nutrition, School of Public Health, Sun Yat-Sen University, Guangzhou 510080, China; zhangjj46@mail2.sysu.edu.cn (J.-J.Z.); liya28@mail2.sysu.edu.cn (Y.L.); 2 Zhongshan Center for Disease Control and Prevention, Zhongshan 528403, China; zscdclsj@163.com; 3 South China Sea Bioresource Exploitation and Utilization Collaborative Innovation Center, Sun Yat-Sen University, Guangzhou 510006, China

**Keywords:** *Sterculia nobilis*, fruit, waste, antioxidant, phenolic compounds, microwave-assisted extraction

## Abstract

The waste of *Sterculia nobilis* fruit was massively produced during food processing, which contains lots of natural antioxidants. In this study, antioxidants in the *Sterculia nobilis* fruit waste were extracted using the green microwave-assisted extraction (MAE) technique. The effects of five independent variables (ethanol concentration, solvent/material ratio, extraction time, temperature, and microwave power) on extraction efficiency were explored, and three major factors (ethanol concentration, extraction time, and temperature) showing great influences were chosen to study their interactions by response surface methodology. The optimal conditions were as follows: 40.96% ethanol concentration, 30 mL/g solvent/material ratio, 37.37 min extraction time at 66.76 °C, and 700 W microwave power. The Trolox equivalent antioxidant capacity value obtained in optimal conditions was in agreement with the predicted value. Besides, MAE improved the extraction efficiency compared with maceration and Soxhlet extraction methods. Additionally, the phenolic profile in the extract was analyzed by UPLC-MS/MS, and eight kinds of phenolic compounds were identified and quantified, including epicatechin, protocatechuic acid, ferulic acid, gallic acid, *p*-coumaric acid, caffeic acid, quercetin, and *p*-hydroxycinnamic acid. This study could contribute to the value-added utilization of the waste from *Sterculia nobilis* fruit, and the extract could be developed as food additive or functional food.

## 1. Introduction

The imbalance between the production of reactive oxygen species and the antioxidant defense system could induce oxidative stress [1,2]. Oxidative stress could cause cell death and tissue damage, and was related to some diseases, including cardiovascular diseases, diabetes, neuronal disorders, and cancer [3]. There is growing scientific evidence that antioxidants could reduce or prevent the negative effects of oxidative stress on living tissues and inhibit aging processes and the development of many diseases [4,5,6]. In addition, antioxidants also play a vital role in the food industry, since they are required for the preservation of biological materials. Synthetic antioxidants such as BHA and BHT have been found very effective in the preservation of foods and in prolonging the shelf life of foods, but are considered to be controversial by consumers. Recently, natural antioxidants have gained increasing attention due to their low toxicity, and they are widely produced by plants including medicinal plants, fruits, and vegetables [7,8,9,10,11,12,13,14,15]. Hence, extraction and identification of natural antioxidants are very necessary and valuable. 

*Sterculia nobilis* belongs to the family of *Sterculiaceae*, and is a tropical woody plant [16]. *Sterculia nobilis* is a valuable medicine for treatment of gastroenteric disorder and bloody flux [17,18]. *Sterculia nobilis* fruit was rich in protein, fiber, vitamins, amino acids, and trace elements. In addition, it contained lots of polyphenols with strong antioxidant activities. The waste of *Sterculia nobilis* fruit was massively produced during food processing, and contains lots of natural antioxidants. Thus, to make good use of waste materials, natural antioxidants in the hull of *Sterculia nobilis* fruit were extracted and identified in this study.

There are several methods that could extract antioxidants from natural materials. Conventional methods included Soxhlet extraction, maceration, and steam distillation, while non-conventional methods included MAE, ultrasound-assisted extraction, subcritical water extraction, and supercritical fluid extraction [19,20,21,22]. Non-conventional methods usually consumed less time and organic solvents than conventional ones. Recently, environmentally friendly and high efficient extraction techniques have been popular. MAE, as a green and efficient extraction method, has gained increasing attention. There are some studies indicating that MAE could reduce extraction time, lower extraction temperature, and achieve a high extraction yield [23,24,25]. The physical principle of the MAE method might be the interaction of materials and microwave energy, as well as the dielectric properties of materials [26,27]. Besides, MAE is maneuverable and inexpensive, and has been selected to extract several bioactive constituents from natural materials [28,29]. Therefore, the MAE method was tested in this study for extracting natural antioxidants from the hull of *Sterculia nobilis*.

Some independent parameters (ethanol concentration, solvent/material ratio, extraction time, temperature, and microwave power) could influence the efficiency of MAE, respectively, and could also have interaction effects [30,31,32,33,34,35,36]. Recently, the response surface methodology (RSM) was utilized frequently for optimizing extraction conditions, because RSM as a mathematical and statistical tool was efficient and reliable [37,38,39]. In this study, the influences of five variables (ethanol concentration, solvent/material ratio, extraction time, temperature, and microwave power) on extraction efficiency were studied respectively via single-factor experiments. Then, RSM was conducted to investigate the interaction of three influential factors. Additionally, MAE was compared with two conventional extraction methods (maceration and Soxhlet extraction). Furthermore, the natural antioxidants in the extract were identified and quantified by UPLC-MS/MS.

## 2. Results and Discussion

### 2.1. Single-Factor Experiment Analysis

#### 2.1.1. Influence of Ethanol Concentration

**Figure 1:**
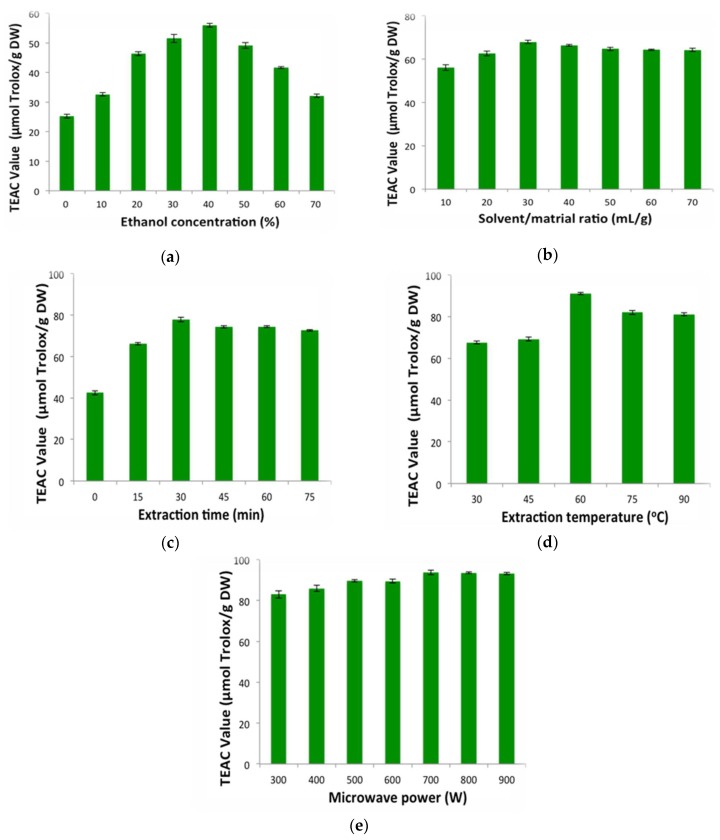
The influence of ethanol concentration (**a**), solvent/material ratio (**b**), extraction time (**c**), temperature (**d**), and microwave power (**e**) on extraction efficiency.

Compared to other organic solvents, aqueous ethanol is safer, cheaper, more easily accessible, and more highly affinitive [40]. Thus, aqueous ethanol was selected as a solvent in this experiment, and toxic methanol and acetone have not been tested in this study. The effect of ethanol concentration on extraction efficiency was studied with solvent/material ratio, 30 mL/g; extraction time, 30 min; temperature, 30 °C; and microwave power, 500 W. It was observed in Figure 1a that Trolox equivalent antioxidant capacity (TEAC) values elevated markedly from 25.16 ± 0.65 to 55.98 ± 1.38 μmol Trolox/g DW, in which ethanol concentration ranged from 0% to 40%. The peak of TEAC value was discovered when ethanol concentration was 40%. Following this, a significant decrease was found with higher ethanol concentration from 50% to 70%. Therefore, 40% ethanol was used in the following experiments.

#### 2.1.2. Influence of Solvent/Material Ratio

Solvent/material ratio (10:1, 20:1, 30:1, 40:1, 50:1, 60:1, and 70:1, mL/g) was chosen to study its impact on the TEAC values of the extract from the hull of *Sterculia nobilis* fruit. Other four variables were designed as follows: 40% ethanol concentration, 30 min at 30 °C, and 500 W microwave power. The data are displayed in Figure 1b. The TEAC values improved gradually when the solvent/material ratio ranged from 10:1 to 30:1. Then, it was almost unchanged with the further solvent/material ratio enhancement from 30:1 to 70:1. Theoretically, a higher solvent/material ratio could provoke bigger concentration difference and promote mass transfer and diffusion [35]. Nevertheless, elevation of solvent/material ratio might have had little influence on extraction efficiency when the mass transfer reached its peak. Similar results were found in the article about extraction of polysaccharides from the *Sargassum thunbergii* [41]. Thus, 30:1 was the optimal solvent/material ratio.

#### 2.1.3. Influence of Extraction Time

The impact of extraction time (0, 15, 30, 45, 60, and 75 min) on the TEAC values of the extract was explored with the following design: 40% ethanol concentration, 30 mL/g solvent/material ratio, and 500 W microwave power at 30 °C. It was found in Figure 1c that the TEAC values promoted markedly from 42.47 ± 0.95 to 77.98 ± 1.19 μmol Trolox/g DW with the extraction time prolonged from 0 to 30 min. However, extraction efficiency increased no more and even decreased a little when the duration of microwave was longer. Obviously, microwave could improve the extraction efficiency in a short duration. However, as the exposure time was longer, microwave caused no promoting effects on extraction efficiency or even might have destroyed the antioxidants. Thus, it is suitable to choose 30 min in the following parts.

#### 2.1.4. Influence of Temperature

Figure 1d displayed the impact of temperature (30, 45, 60, 75, and 90 °C) on TEAC values of the sample, which was studied with 40% ethanol concentration, 30 mL/g solvent/material ratio, 30 min extraction time, and 500 W microwave power. It could be observed in Figure 1d that TEAC values increased significantly from 67.63 ± 0.73 to 91.16 ± 0.57 μmol Trolox/g DW with an elevation of the temperature (30 to 60 °C). This phenomenon could be attributed to the diffusion accelerated by suitable high temperature. Following this, a falling trend of extraction efficiency appeared. The reason for this trend might be thermolabile antioxidants decomposed by high temperature (>60°C) [40]. Consequently, 60 °C was the optimal temperature.

#### 2.1.5. Influence of Microwave Power

Microwave power was another important factor affecting extraction efficiency. Different microwave power (300, 400, 500, 600, 700, 800, and 900 W) was studied with four variables designed as 40% ethanol concentration, 30 mL/g solvent/material ratio, and 30 min at 60 °C. According to Figure 1e, the yield of antioxidants presented an uptrend, as the microwave power increased (300 W to 700 W). The TEAC values achieved the maximum (92.67 ± 0.46 μmol Trolox/g DW), while the microwave was 700 W. When microwave power exceeded 700 W, the antioxidant activity slightly decreased. This fact indicated that extraction efficiency could be improved by microwave exposure via accelerating the movement of solvents, diffusion of antioxidants, and breaking of cell wall. Besides, microwave had negative effects of inducing degradation of antioxidants [42,43]. When microwave power was not extremely high, beneficial effects of microwave were greater than negative effects, and extraction efficiency could be elevated. Hence, 700 W was the suitable microwave power.

### 2.2. RSM Analysis

#### 2.2.1. Central Composite Rotatable Design (CCRD) and Results

RSM using CCRD design was chosen for further optimization of the extraction efficiency of antioxidants from the hull of *Sterculia nobilis*. Three parameters (ethanol concentration, extraction time, and temperature) that made greater influences on extraction efficiency than other factors were selected. The middle level of these factors was 40% ethanol concentration, 30 min extraction time, and 60 °C temperature, which were obtained from the single-factor experiments. Besides, the other two factors were controlled as 30 mL/g solvent/material ratio and 700 W microwave power. The data were displayed in the Table 1, which included 20 runs experiment designs, actual values, and corresponding predicted values. The data showed that the actual TEAC values varied from 46.08 to 94.25 μmol Trolox/g DW.

#### 2.2.2. Fitting the Model

A quadratic regression equation was obtained by analysis of the data in Table 1. Equation (1) showed the combined influences of three parameters (ethanol concentration (X_1_), extraction time (X_2_), and temperature (X_3_)) on TEAC values (Y).

Y = −417.92 + 5.05X_1_ + 3.04X_2_ + 10.56X_3_ − 0.0023X_1_X_2_ − 0.0023X_1_X_3_ − 0.0088X_2_X_3_ − 0.059X_1_^2^ − 0.032X_2_^2^ − 0.076X_3_^2^ (1)

Analysis of variance (ANOVA) was used to examine the reliability of the fitting model. As shown in Table 2, the high F value (10.06) and very low *p* value of 0.0006 (<0.001) of the model indicated that this regression model was credible. Besides, low F value of 0.35 and high *p* value of 0.8657 suggested that “lack of fit” was not significant, which further confirmed that the model was valid and able to predict the variation precisely. Additionally, according to determination coefficient (R^2^) (0.9006) and the adjusted R^2^ (0.8110), the regression model could elucidate almost 81.10% of the response value variations. For the three parameters, both the linear and quadratic effects of extraction time (X_2_) and temperature (X_3_) were significant (*p* < 0.05) on the TEAC values, while the ethanol concentration (X_1_) only presented a significant (*p* < 0.05) quadratic effect.

#### 2.2.3. Response Surfaces Analysis

**Figure 2:**
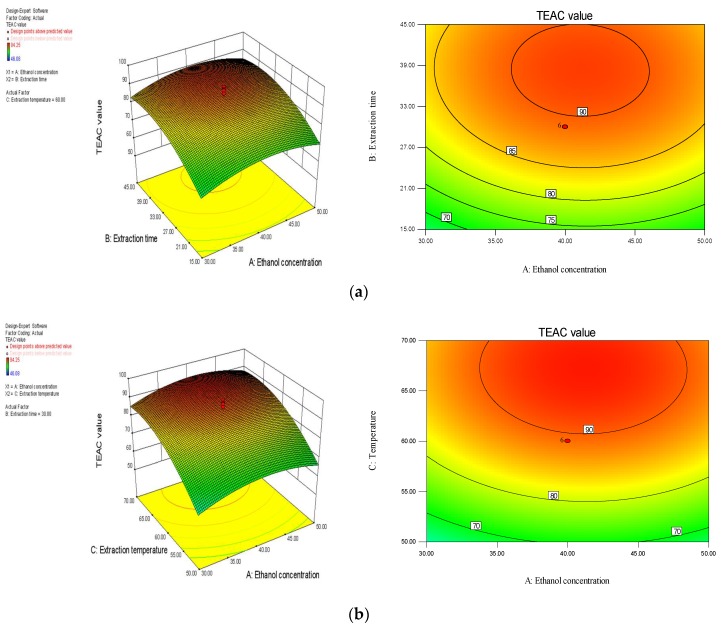

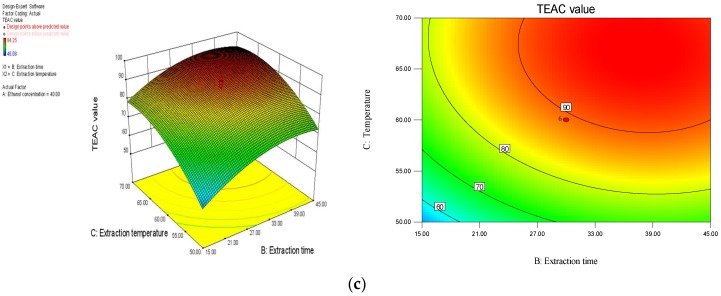
Interaction effects of ethanol concentration (%) and extraction time (min) (**a**); ethanol concentration and temperature (°C) (**b**); and extraction time and temperature (**c**) on TEAC value (μmol Trolox/g DW).

The interaction between the response value (Y, TEAC value) and major parameters (X_1_, ethanol concentration; X_2_, extraction time; X_3_, temperature) can be seen directly from the three-dimensional response surface plots and contour plots. As displayed in Figure 2a, the response value improved with the longer extraction time, when the temperature was 60 °C. However, as the extraction time exceeded 38 min, a decrease of response value was found. Besides, the increasing ethanol concentration (from 30% to 40%) induced an increase of response value. It was observed that the extraction time had a greater influence on the TEAC value than ethanol concentration. In Figure 2b, when the extraction time was set as 30 min, initial increase of extraction temperature (from 50 °C to 67 °C) exerted a great elevation of response value. Then, the response value decreased with the further elevation of temperature. The impact of ethanol concentration on the response value in Figure 2b was similar to that seen in Figure 2a. In Figure 2c, ethanol concentration was fixed at 40%, and the interaction between the extraction time and temperature on the TEAC value was elucidated. The effects of the extraction time and temperature on the TEAC value were similar to those in Figure 2a,b. It was found that the temperature showed a greater impact on the TEAC value than the ethanol concentration and extraction time, according to the analysis of ANOVA and the response surface plots.

#### 2.2.4. Validation of Predicted Value

The most suitable microwave extraction conditions in the study were as follows: 40.96% ethanol concentration, 30 mL/g solvent/material ratio, 37.37 min extraction time at 66.76 °C, and 700 W microwave power, which were obtained by analyzing the model. The predicted value of the highest TEAC was 94.88 μmol Trolox/g DW according to the model. The validation experiment under the optimal conditions was conducted to examine the reliability of the model. The TEAC actual value of the verification experiment was 93.72 ± 1.05 μmol Trolox/g DW, which corresponded closely to the predicted value. Therefore, it was reliable to use RSM for optimizing extraction efficiency. 

### 2.3. Comparison of MAE with Conventional Methods

In this part, a comparison of MAE with two conventional extraction methods including maceration method and Soxhlet extraction was carried out. As shown in Table 3, in terms of extraction efficiency, the antioxidant activity by MAE (93.72 ± 1.05 μmol Trolox/g DW) was 2.24 times and 3.93 times that acquired by maceration method (41.92 ± 1.96 μmol Trolox/g DW) and Soxhlet extraction (23.84 ± 3.06 μmol Trolox/g DW), respectively. In terms of extraction time, MAE (37.37 min) required significantly less time than maceration method (24 h) and Soxhlet extraction (4 h). Besides, the temperature of Soxhlet extraction (95 °C) was also greatly elevated compared to MAE (66.76 °C). Microwave power polarized the polar molecules and the ionic species, which induced fast volumetric heating via dipolar polarization and ionic conduction and increased reaction rates [27]. This might be the reason for the highest efficiency of MAE among the three approaches. The consequences were similar to two reports related to blueberry leaves and *Gordonia axillaris* [44,45]. 

### 2.4. Analysis of Phenolic Compounds

**Figure 3:**
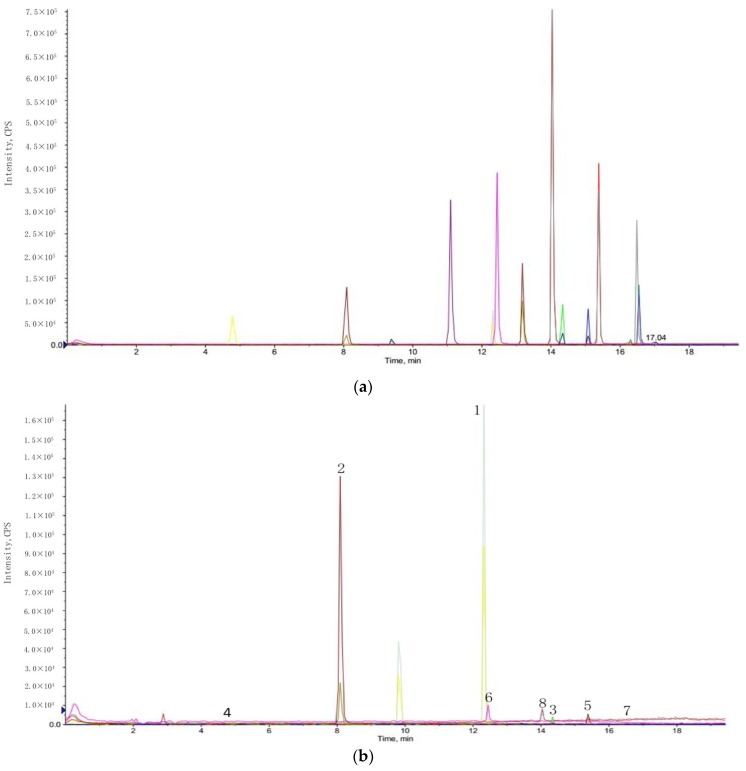
The total ion chromatograms of standard phenolic components (**a**) and the extract acquired under the optimal conditions (**b**).

Phenolic compounds showed strong antioxidant activity, and the identification of these components was helpful to understand the antioxidant properties of the extract [46,47]. In this part, the phenolic compounds in the hull of *Sterculia nobilis* fruit were identified by UPLC-MS/MS. Figure 3 displayed the total ion chromatograms of standard phenolic components and the extract acquired under the optimal conditions. Table 4 showed eight phenolic constituents that existed in the sample. The content of epicatechin was the highest, followed by protocatechuic acid, ferulic acid, gallic acid, *p*-coumaric acid, and caffeic acid. The antioxidant capacity of the hull of *Sterculia nobilis* fruit might be attributed to these phenolic components synergistically. In addition, these phenolic components also had other bioactivities, including antibacterial, anti-inflammatory, and anticancer effects [48,49,50]. Hence, the hull of *Sterculia nobilis* fruit might possess similar bioactivities and potential health benefits.

## 3. Materials and Methods

### 3.1. Chemicals and Reagents

Trolox (6-hydroxy-2,5,7,8-tetramethylchromane-2-carboxylic acid) and ABTS (2,20-azinobis(3-ethyl-benothiazoline-6-sulphonic acid) diammonium salt) were purchased from Sigma-Aldrich (St. Louis, MO, USA). Potassium persulphate was obtained from Tianjin Chemical Factory (Tianjin, China). Ethanol and methanol were purchased from Kelong Chemical Factory (Chengdu, China). Chemicals and reagents used in the study were analytically pure and deionized water was used.

### 3.2. Instruments

The MAE was conducted in an X-100A microwave extraction device (Xianghu Instrumental Company, Beijing, China) with a 1000 W microwave power, which had temperature monitor and microprocessor programmer software that could regulate experiment variables including extraction temperature, time, and microwave power.

### 3.3. Sample Preparation 

The hull of *Sterculia nobilis* fruit was collected and dried at room temperature. Then, the hull was ground into fine particles that were smaller than 0.300 mm by a food grinder (RHP-100, Ronghao Industry & Trade Co. Ltd., Yongkang, China) and was stored at 4 °C in a refrigerator until utilized.

### 3.4. Extraction of Antioxidants

#### 3.4.1. Microwave-Assisted Extraction

The ground powder of the hulls of *Sterculia nobilis* fruit (0.05 g) was put in a capped tube. Proper aqueous ethanol was added to the powder. The mixture was shaken for 5 min in vortex mixer to soak the mixture completely. Then, the sample was placed into a water bath of the microwave device. After irradiation with the pre-set parameters (extraction time, temperature, and microwave power), the tube containing the sample was centrifuged at 4200 *g* for 15 min, and the TEAC value of supernatant was determined.

#### 3.4.2. Maceration Extraction

Firstly, the ground powder of the hulls (0.05 g) was placed in a capped tube. Then, 15 mL of 40.96% ethanol was added to the powder. After 24 h maceration in a shaking water bath at 25 °C, the mixture in the tube was centrifuged at 4200 *g* for 15 min, and the supernatant was collected for subsequent determination. 

#### 3.4.3. Soxhlet Extraction

The Soxhlet extraction referred to the experimental process designed by Xu et al. [51]. The ground powder of the hulls (2.0 g) was wrapped by Whatman filter paper, with 400 mL of 40.96% ethanol in Soxhlet extractor. After 4 h extraction at 95 °C, the extraction solution was obtained for next analysis.

### 3.5. Determination of Antioxidant Capacity

There are several methods that could evaluate the antioxidant capacity of the sample, but the results gained using different methods generally have a very high correlation [7,12]. The TEAC assay was frequently used for evaluation of antioxidant capacity owing to its simplicity and rapidness [7]. The antioxidant activity of the extract was evaluated by TEAC assay according to previous procedure [52]. Tersely, the ABTS^•+^ stock solution was prepared by mixing potassium persulfate (2.45 μmol/L) and ABTS^•+^ (7 μmol/L) with a volume ratio of 1:1. Then, the ABTS^•+^ stock solution was incubated for 16 h in dark environment at room temperature and was utilized within 48 h. The ABTS^•+^ stock solution was diluted to insure that the absorbance of ABTS^•+^ working solution was 0.70 ± 0.05 at 734 nm. Finally, 3.8 mL ABTS^•+^ working solution was added to 100 μL diluted sample, and the absorbance was determined at 734 nm after incubation 6 min in dark environment at normal temperature. The results were expressed as μmol Trolox/g DW.

### 3.6. Determination of Total Phenolic Content

The method of determination of total phenolic content (TPC) in the extract was referred to the previous article [13]. The gallic acid was employed for reference standard, and the results were stated as mg gallic acid equivalent (GAE)/g DW.

### 3.7. Determination of Total Flavonoid Content 

The total flavonoid content (TFC) in the extract was measured according to the established process [53]. The quercetin was selected as the reference standard, and the results were stated as mg quercetin equivalent (mg QE)/g DW.

### 3.8. Identification and Quantification of Phenolic Compounds

The phenolic constituents in the sample acquired under optimal conditions were evaluated referring to the procedure established by Zhou et al. with a little modification [52]. Phenolic compounds were identified and quantified by AB Sciex 4000 Qtrap liquid chromatography-tandem mass spectrometry (SCIEX, Framingham, MA, USA), and Acquity UPLC^®^ HSS T3 column (3.0 × 150 mm, 1.8 μm, Waters, Milford, MA, USA) was used to separate at 40 °C. Solution A (0.2% formic acid aqueous solution) and solution B (methanol) formed the mobile phase, and the flow rate was 0.3 mL/min. The gradient elution was carried out as follows: 0–2 min, 15% (B); 2–8 min, 15–30% (B); 8–15 min, 30–80% (B); 15–17.5 min, 80% (B); and 17.5–19.5 min, 15% (B). The sample was injected with 2 μL. The parameters of mass spectrometry were controlled as follows: ESI source with negative mode, ion source temperature at 550 °C, 4500 V capillary voltage with the mode of multiple reaction monitoring and 10 psig curtain gas, and 20 psig nebulizer gas and 20 psig auxiliary gas. Firstly, the identification of phenolic constituents was conducted by tandem mass spectrometry. Then, verification and quantification of these constituents were acquired by contrasting the retention times and peak areas of corresponding standards.

### 3.9. Experiment Design

#### 3.9.1. Single-Factor Experiments 

Five variables, including ethanol concentration, solvent/material ratio, extraction time, temperature, and microwave power, were selected to evaluate their effects on antioxidant capacity of the extract from the hull of *Sterculia nobilis* fruit. The levels of each factor were designed as follows: ethanol concentration 20% to 70%, solvent/material ratio 10:1 to 70:1, extraction time 0 to 75 min, temperature 30 to 90 °C, and microwave power 300 to 900 W. Three factors that made significant differences on extraction efficiencies would be chosen to conduct subsequent experiments.

#### 3.9.2. Response Surface Methodology

RSM using CCRD was used to further optimize the antioxidants capacity of the extract from the hull of *Sterculia nobilis* fruit. As shown in Table 5, three key variables (ethanol concentration (X_1_), extraction time (X_2_), and temperature (X_3_)) were selected and evaluated in 5 levels. 20 experimental runs including 6 replicates in the central point were carried out. The data of CCRD were based on a second-order polynomial model, as follows:

Y = β_0_ + ∑β_i_X_i_ + ∑β_ii_X_i_^2^ + ∑β_ij_X_i_X_j_ (2)

#### 3.9.3. Statistical Analysis

All the experiments were conducted in triplicate. The average value ± SD (standard deviation) was reported. Statistical analysis was implemented using Design Expert 8.0.6 and Excel 2016.

## 4. Conclusions

An environmentally friendly MAE method has been established to extract antioxidants from the hulls of *Sterculia nobilis* fruit, and RSM was selected to further optimize three experimental factors. The results indicated that the optimal extraction conditions were 40.96% ethanol concentration, 30 mL/g solvent/material ratio, 37.37 min at 66.76 °C, and 700 W microwave power. Under the optimal conditions, the maximum TEAC value obtained was 93.72 ± 1.05 μmol Trolox/g DW, and TPC, as well as TFC of the extract, was 3.67 ± 0.80 and 0.45 ± 0.13 μmol Trolox/g DW, respectively. The high R^2^ and consistency between the predicted and actual value demonstrated the preciseness and reliability of the model. Additionally, MAE was more efficient at extracting antioxidants from the hulls of *Sterculia nobilis* fruit compared to two conventional methods. In conclusion, MAE is a promising technique for natural constituents extraction because of its high efficiency and environmentally friendly character. Finally, epicatechin, protocatechuic acid, ferulic acid, gallic acid, *p*-coumaric acid, caffeic acid, quercetin, and *p*-hydroxycinnamic acid were identified and quantified, which might contribute greatly to the antioxidant activity of the hulls of *Sterculia nobilis* fruit. This study could be helpful for the value-added utilization of the waste from *Sterculia nobilis* fruit, and the extract could be developed as food additive or functional food.

## Figures and Tables

**Table 1:** The experimental design and results of RSM.

Run	X1 (Ethanol Concentration, %)	X2 (Extraction Time, min)	X3 (Temperature, °C)	Y (TEAC Value, μmol Trolox/g DW)	
				Actual Value	Predicted Value
1	40	55.23	60	80.50	82.38
2	40	30	60	92.64	89.16
3	30	45	70	88.26	85.23
4	30	15	50	46.08	46.38
5	30	15	70	76.50	71.37
6	40	30	76.82	80.73	86.10
7	40	30	60	94.25	89.16
8	23.18	40	60	65.21	70.08
9	30	45	50	67.75	65.55
10	40	30	60	93.10	89.16
11	50	45	70	89.64	87.01
12	40	30	60	90.79	89.16
13	40	4.77	60	54.38	55.78
14	50	45	50	65.44	68.25
15	40	30	60	92.41	89.16
16	50	15	70	74.66	74.54
17	40	30	43.18	51.39	49.31
18	40	30	60	72.36	89.16
19	56.82	30	60	76.60	75.02
20	50	15	50	49.77	50.48

**Table 2:** ANOVA for the response surface model.

Source	Sum of Squares	df	Mean Square	F Value	*p* Value	Significant
Model	4252.35	9	472.48	10.06	0.0006	significant
X_1_	29.51	1	29.51	0.63	0.4463	
X_2_	854.21	1	854.21	18.19	0.0016	
X_3_	1633.58	1	1633.58	34.79	0.0002	
X_1_X_2_	0.97	1	0.97	0.021	0.8888	
X_1_X_3_	0.42	1	0.42	0.009	0.9262	
X_2_X_3_	14.04	1	14.04	0.30	0.5964	
X_1_^2^	497.42	1	497.42	10.59	0.0087	
X_2_^2^	726.50	1	726.50	15.47	0.0028	
X_3_^2^	829.77	1	829.77	17.67	0.0018	
Residual	469.59	10	46.96			
Lack of Fit	120.65	5	24.13	0.35	0.8657	not significant
Pure Error	348.94	5	69.79			
Cor Total	4721.94	19				
R-Squared	0.9006					
Adj R-Squared	0.8110					

**Table 3:** The comparison of MAE with maceration and Soxhlet extraction.

Extraction Methods	Ethanol Conc. (%)	Extraction Time (min)	Temp. (°C)	TEAC (μmol Trolox/g DW)	TPC (mg GAE/g DW)	TFC (mg QE/g DW)
Maceration	40.96	24 h	25	41.92 ± 1.96	2.74 ± 0.69	0.30 ± 0.17
Soxhlet	40.96	4 h	95	23.84 ± 3.06	2.56 ± 0.64	0.24 ± 0.10
MAE	40.96	37.37	66.76	93.72 ± 1.05	3.67 ± 0.80	0.45 ± 0.13

**Table 4:** The contents of phenolic compounds in the extract acquired under the optimal condition.

Number	Phenolic Compounds	Retention Time (t_R_, min)	Paront Ion (*m*/*z*, [M − H]^−^)	Product Ion (*m*/*z*)	Contents (μg/g DW)
1	Epicatechin	12.3	289	203	56.63 ± 0.58
2	Protocatechuic acid	8.08	153.1	109	21.09 ± 0.16
3	Ferulic acid	14.3	193.1	134	0.84 ± 0.003
4	Gallic acid	4.79	169.1	125	0.53 ± 0.008
5	*p*-Coumaric acid	15.4	162.7	119	0.45 ± 0.003
6	Caffeic acid	12.4	179.1	135	0.35 ± 0.010
7	Quercetin	16.5	301	179	0.041 ± 0.001
8	*p*-Hydroxycinnamic acid	14	163.1	119	0.027 ± 0.001

**Table 5:** Five variables and their levels of CCRD.

Variable	Units	Symbol	Code Levels				
			−1.68	−1	0	1	1.68
Ethanol concentration	% (*v*/*v*)	X_1_	23.18	30	40	50	56.82
Extraction time	min	X_2_	4.77	15	30	45	55.23
Temperature	°C	X_3_	43.18	50	60	70	76.82
